# Engineering CRISPR/Cas9 therapeutics for cancer precision medicine

**DOI:** 10.3389/fgene.2024.1309175

**Published:** 2024-04-25

**Authors:** Aditya Kumar Sharma, Anil K. Giri

**Affiliations:** ^1^ Department of Pathology, College of Medicine, University of Illinois at Chicago, Chicago, IL, United States; ^2^ Institute for Molecular Medicine Finland, University of Helsinki, Helsinki, Finland; ^3^ Foundation for the Finnish Cancer Institute, Helsinki, Finland

**Keywords:** CRISPR/Cas9, cancer treatment, preclinical studies, precision medicine, CRISPR/Cas9 therapeutics

## Abstract

The discovery of Clustered Regularly Interspaced Short Palindromic Repeats (CRISPR) and CRISPR-associated protein 9 (Cas9) technology has revolutionized field of cancer treatment. This review explores usage of CRISPR/Cas9 for editing and investigating genes involved in human carcinogenesis. It provides insights into the development of CRISPR as a genetic tool. Also, it explores recent developments and tools available in designing CRISPR/Cas9 systems for targeting oncogenic genes for cancer treatment. Further, we delve into an overview of cancer biology, highlighting key genetic alterations and signaling pathways whose deletion prevents malignancies. This fundamental knowledge enables a deeper understanding of how CRISPR/Cas9 can be tailored to address specific genetic aberrations and offer personalized therapeutic approaches. In this review, we showcase studies and preclinical trials that show the utility of CRISPR/Cas9 in disrupting oncogenic targets, modulating tumor microenvironment and increasing the efficiency of available anti treatments. It also provides insight into the use of CRISPR high throughput screens for cancer biomarker identifications and CRISPR based screening for drug discovery. In conclusion, this review offers an overview of exciting developments in engineering CRISPR/Cas9 therapeutics for cancer treatment and highlights the transformative potential of CRISPR for innovation and effective cancer treatments.

## Introduction

Cancer is a prime cause of death globally. It is a convoluted disease involving changes in genome (e.g., addition, deletion, single-nucleotide change) and epigenomes, which leads to change gene expression involved in carcinogenesis (Sharma et al., 2010; Tomczak et al., 2015). These changes lead to abnormal cell growth unlike the normal healthy cell division, which is tightly regulated and only occurs when instructed. It affects cells and thrives on modifications to metabolism, cell structure and motility to promote growth in unfavorable environments (Mercadante and Kasi, 2023). Over the decades, researchers have identified many genes and regulatory pathways whose dysregulation leads to cancer development in the body (Table 1). However, these cancer-driving genomic and epigenomic changes in cancer cells are specific across patients and can show intra- and inter tumor heterogeneity even within a patient (Sun and Yu, 2015). This heterogeneity affects cancer progression, adaptations to external change in microenvironment, response to therapeutic assault and contributes to drug resistance (Ge et al., 2022). Understanding the effect of these cancers causing changes in individual patients is crucial for developing new therapeutic treatments. Tools that can help to study the impact of these genetic changes on cancer growth and survival either in preclinical or patients can help to better treat the disease in a personalized way.

**TABLE 1:** Major oncogenes and associated cancers. The resources have been obtained from www.cancer.gov.

S.NO	Gene	Name	Major cancer associated (%)
1	BRCA1, BRCA2	Breast Cancer gene 1	Breast, ovarian, prostate pancreatic, colon cancer
2	PTEN	Phosphatase and tensin homolog	Breast, renal cell carcinoma, prostate, colorectal, melanoma, glial and lung cancer
3	TP53	Tumor protein p53	Ovarian, colorectal, breast, head and neck, lung, melanoma, liver, brain and other type of cancers
4	EGFR	Epidermal growth factor receptor	Lung, glioblastoma, colorectal, pancreatic, breast cancer
5	KRAS	Kirstan rat sarcoma viral oncogene homolog	Lung, colorectal, pancreatic, and Testicular germ cell cancer
6	APC	Adenomatous polyposis coli	Colorectal cancer, lung cancer
7	HER2	Human epidermal growth factor receptor 2	Breast, bladder, pancreatic, ovarian, gastric cancer
8	CDKN2A	Cyclin dependent kinase inhibitor 2A	Lung, melanoma, pancreatic, glioblastoma cancer
9	RB1	Retinoblastoma 1 transcriptional corepressor 1	Retinoblastoma, lung cancer
10	VHL	Von Hippel Lindau syndrome	Renal cell carcinoma, lung, colon, pancreatic cancer
11	RET	Ret proto-oncogene	Thyroid, lung, colon, melanoma
12	NF1	Neurofibromatosis type 1	Malignant peripheral nerve sheath tumor, Lung
13	BCR-ABL1	BCR-Abelson murine leukemia viral oncogene homolog1	Chronic myeloid leukemia, breast cancer
14	FLT3	Fms related receptor tyrosine kinase 1	Acute myeloid leukemia, colon adenocarcinoma, lung adenocarcinoma, cutaneous melanoma, breast invasive ductal carcinoma
15	JAK2	Janus Kinase 2	Myeloproliferative neoplasm, lung adenocarcinoma, colon adenocarcinoma, breast invasive ductal carcinoma, polycythemia vera
16	CEBPA	CCAAT enhancer binding protein alpha	Acute myeloid leukemia, lung adenocarcinoma, colon adenocarcinoma, breast invasive ductal carcinoma
17	MDM2	Murine double minute 2	Sarcoma, liposarcoma, lung adenocarcinoma, breast invasive ductal carcinoma, glioblastoma multiforme
20	ALK	Anaplastic lymphoma kinase	Lung adenocarcinoma, neuroblastoma, pancreatic adenocarcinoma
21	IDH1	Isocitrate dehydrogenase 1	Glioblastoma multiforme, acute myeloid leukemia, anaplastic astrocytoma, oligodendroglioma
22	NOTCH1	Neurogenic locus notch homolog protein 1	T-cell acute lymphoblastic leukemia, lung adenocarcinoma, colon adenocarcinoma, breast invasive ductal carcinoma, small lymphocytic lymphoma, skin squamous cell carcinoma
23	SMAD4	SMA- and MAD-related protein 4	Pancreatic adenocarcinoma, colorectal adenocarcinoma, lung adenocarcinoma, rectal adenocarcinoma
24	PIK3CA	Phosphotidylinositol-4-5-biphosphate 3-kinase catalytic subunit alpha	Breast, colon adenocarcinoma, colorectal adenocarcinoma, endometrial endometrioid adenocarcinoma, lung adenocarcinoma
25	AKT1	AKT serine/threonine kinase 1	Breast, colon adenocarcinoma, colorectal adenocarcinoma, endometrial endometrioid adenocarcinoma, lung adenocarcinoma
26	BCL2	B cell leukemia/lymphoma 2 protein	B-cell lymphoma, leukemia
27	TSC1	Tuberculosis sclerosis complex 1	Tuberous sclerosis, renal cell carcinoma, endometrial endometrioid adenocarcinoma
28	HNF1A	Hepatocyte nuclear factor 1 alpha	Liver, pancreatic cancer, renal cell carcinoma, colon and lung adenocarcinoma
29	PDGFRA	Platelet derived growth factor receptor A	Gastrointestinal tumors, melanoma, glioblastoma multiforme, lung adenocarcinoma
30	STK11	STK11 serine/threonine kinase 11	Lung cancer
31	SMARCB1	SWI/SNF-related matrix associated actin dependent regulator of chromatin subfamily B member 1	Rhabdoid tumor, colon and lung adenocarcinoma, endometrial endometrioid adenocarcinoma
32	PTCH1	Protein patched homolog 1	Basal cell carcinoma, medulloblastoma
33	KIT	KIT proto-oncogene receptor tyrosine kinase	Gastrointestinal stromal tumors, melanoma, colon and lung adenocarcinoma
34	CDH1	Cadherin 1	Gastric, breast cancer, colon and lung adenocarcinoma
35	MEN1	Multiple endocrine neoplasia link type 1	Multiple endocrine neoplasia, breast cancer

CRISPR-associated protein 9 (Cas9) systems provide one-such unique tool that enables not only to edit (e.g., add, delete, substitute) the genome but also allows transcriptional and epigenome apparatus using dead Cas9D10A/H840A (dCas9), which is incapable of cleaving DNA (Wang et al., 2016; Brezgin et al., 2019). Similarly, the technique known as CRISPR interference (CRISPRi) is used to block the transcription of target genes by combining the specific DNA recognition dCas9 with the Kruppel-associated box (KRAB) repressor (Thakore et al., 2015). In a similar approach, to induce robust gene induction at target location, dCas9 is attached to transcriptional activators like VP64 and VP64–p65–Rta (VPR) proteins (Lo and Qi, 2017). According to studies, fusing dCas9 with either (DNA methyltransferase 3A) or PRDM9 (PR domain-containing protein 9), both of which are methyltransferases, can provide insights into DNA’s epigenetic regulation. Additionally, fusing dCas9 with demethylation enzymes like TET (tet methylcytosine dioxygenase1) or LSD1/KDM1A (lysine-specific histone demethylase 1) can also aid in exploring DNA’s epigenetic regulation (O'Geen et al., 2017; Stepper et al., 2017; Chen et al., 2022; Choudhury et al., 2016; Lau and Suh, 2018; Katti et al., 2022).

## Brief history of CRISPR/Cas9 development

James Watson and Francis Crick’s discovery of the DNA double helix in 1953 provided researchers with a fundamental understanding of the structure and functions of genetic material (Watson and Crick, 1953). This breakthrough laid the foundation of modern molecular medicine and understanding the role of the smallest unit of genetic information called genes (Avery et al., 1944; Strasser, 2003). With the advancement of molecular tools in 1979, Scherer et al. published a method that can introduce the foreign DNA sequence *in vitro* to *Saccharomyces cerevisiae* chromosomal DNA (Scherer and Davis, 1979). Similarly, the integration of plasmid into the human globin locus using homologous recombination was also demonstrated by Smithies et al. (Smithies et al., 1985). In 1988, Mansour et al. showed foreign DNA can introduce to mouse embryonic stem cells to disrupt a proto-oncogene int-2, which suggests that any gene in the genome can be disrupted (Mansour et al., 1988). In the 1980s, gene targeting methodology was based on DNA repair and DNA base pair recognition, which enabled scientists to make precise changes in the genome (Batty and Wood, 2000; Rajski et al., 2000; Wood et al., 2000; Dalhus et al., 2009). The other methods that have been employed over time include zinc finger nucleases, TAL effector nucleases, peptide nucleic acids, and polyamides for efficient DNA cleavage and inducing a change in DNA sequence (Good and Nielsen, 1999; Nielsen and Egholm, 1999; Cathomen and Joung, 2008; Simon et al., 2008; Christian et al., 2010; Li and Yang, 2013; Koeller et al., 2014; Gaj et al., 2016; Yu et al., 2019). However, these methods have their own limitations, such as complex designing, inefficient delivery, potential toxicity, expensive, and possible off-target effects (Gaj et al., 2016; Montazersaheb et al., 2018; Lin and Nagase, 2020; Gonzalez Castro et al., 2021). CRISPRs were first described by Ishino et al., in 1987 as short interspersed sequences in the genome of *Escherichia coli* while investigating the gene "alkaline phosphatase” (Ishino et al., 1987). Mojica et al. found repetitive sequences in prokaryotic genome, which named CRISPRs (Mojica et al., 1993; Mojica et al., 2005; Ishino et al., 2018). Later, many studies have shown that CRISPRs have short spacers that are derived from foreign genetic material, and CRISPRs provide adaptive resistance against viruses (Makarova et al., 2006; Barrangou et al., 2007; Brouns et al., 2008; Hale et al., 2009). Barrangou et al. have demonstrated the incorporation of spacer sequence from the phage genome into S. thermophilus chromosome upon viral challenge (Barrangou et al., 2007). Studies speculate the involvement of some Cas genes in the insertion of sequences and providing resistance against the viruses (Makarova et al., 2006; Ibrahim et al., 2019). Based on this information, Brouns et al. investigated how bacterial Cas proteins employ virus-derived sequences in CRISPRs to provide antiviral resistance (Brouns et al., 2008). In order to prevent viral replication in *E.coli*, it was demonstrated that mature CRISPR RNAs serves as guide to bacterial Cas protein (Brouns et al., 2008). Marraffini et al. later showed CRISPR/Cas system in *S. epidermidis* prevents the horizontal transfer of plasmid and indicates that CRISPR/Cas machinery targets DNA directly (Marraffini and Sontheimer, 2008). Therefore, these spacers function as a memory of the previous infection and protect bacteria from subsequent virus attacks. Many studies have suggested CRISPR/Cas9 as an accurate and effective tool for DNA editing (Hale et al., 2009; Bortesi and Fischer, 2015; Ryan et al., 2016; Zhu et al., 2017; Uyhazi and Bennett, 2021). Finally, Doudna, J., and Charpentier, E. discovered in 2012 that CRISPR/Cas-9 can be utilized for editing DNA sequences and won 2020 Noble Prize for Chemistry for their work in developing CRISPR technology (Westermann et al., 2021).

## CRISPR/Cas9 components and its mechanism

**FIGURE 1:**
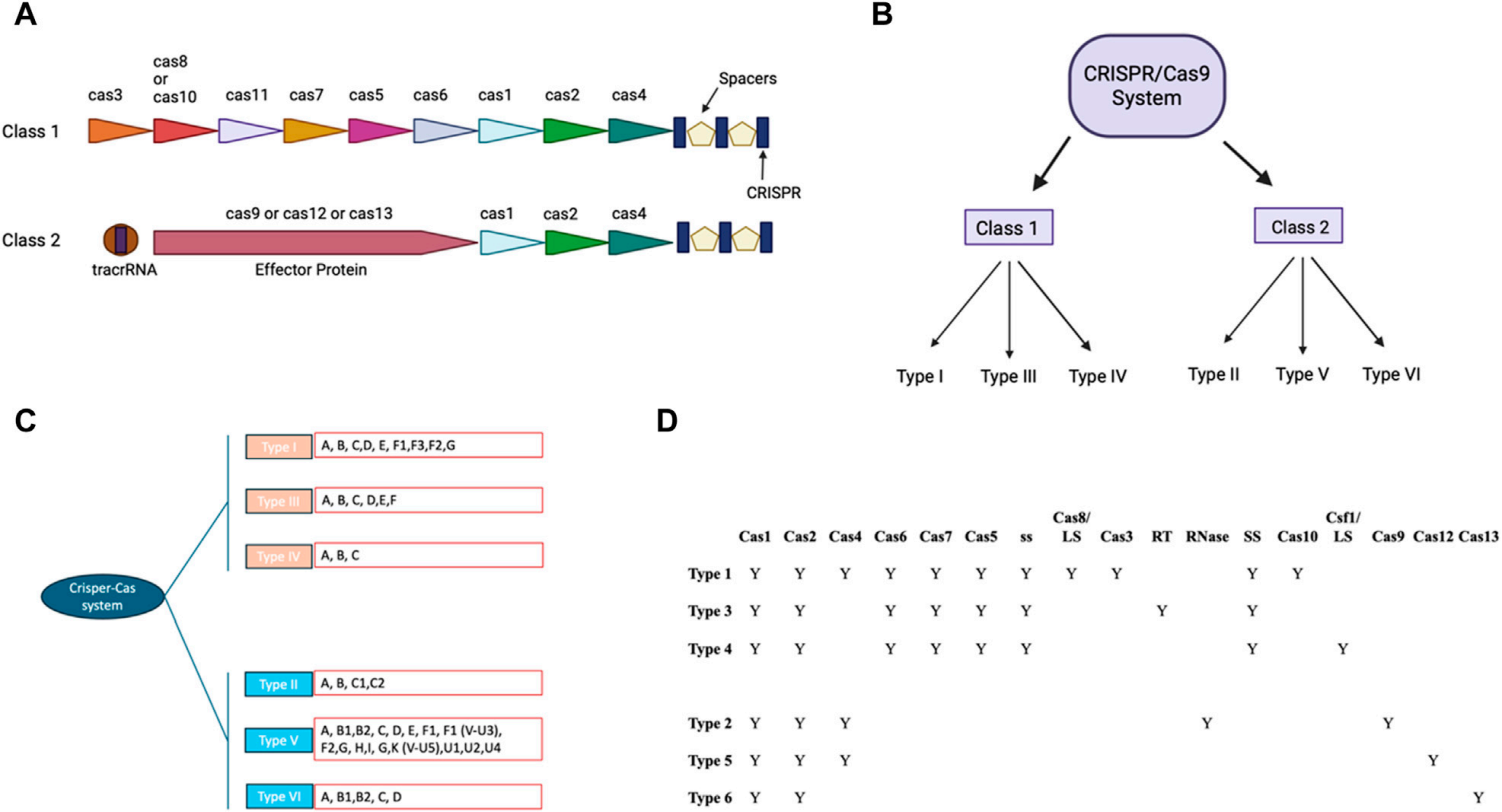
CRISPR-Cas: Two classes and their modular organization **(A)** Visual representation showing organization of Class I and II. **(B)** Classification of CRISPR/CAS9 system in different Class. **(C)** Classification of CRISPR/CAS9 system to types and subtypes: The different subtypes of the traditional type 1 CRISPR/CAS9 system have been colored pink and the type 2 CRISPR/CAS9 system has been colored in blue. The different types of these subtypes have been written in the red box. **(D)** Tabular representation of different cas9 and other domains present in different subtypes of class of CRISPR/Cas9 (with BioRender.com).

CRISPR/Cas9 system includes guides RNA that help DNA endonuclease Cas9 cause double-stranded breaks at specific site in the DNA sequence (Ran et al., 2013). crRNA and tracrRNA are the two RNA components of guides present in CRISPR/Cas9 system (Koonin and Makarova, 2009; Wang et al., 2016). Both of these RNA guides have different functions, as 20–22 bp crRNA binds with desired DNA sequence while tracrRNA acts as a framework for Cas9 nuclease that causes DNA breaks (Koonin and Makarova, 2009; Wang et al., 2016). There are six types of CRISPR systems (I-VI), each of them employs a specific set of Cas proteins and guide RNA to modify the genome (Jiang and Doudna, 2017; Koonin et al., 2017) (Figure 1). The CRISPR Type II system is the extensively studied CRISPR/Cas9 system that involves a single DNA endonuclease, Cas9 (Xu and Li, 2020). On the other hand, CRISPR Type I and II systems use multiple-Cas proteins complex for guide RNA binding and targeted blunt-end double-strand DNA breaks (Jiang and Doudna, 2017). Cas9 derived from *S. pyrogenes* is also referred to as SpCas9 (Jiang and Doudna, 2017). It is a multifunctional DNA endonuclease having 1,368 amino acids with two distinct regions (Mei et al., 2016). The two distinct regions of Cas9 are recognition lobe and nuclease lobe, which are responsible for binding guide RNA to desired DNA region and double strand break of DNA sequence, respectively (Nishimasu et al., 2014). The nuclease lobe of CRISPR/Cas9 consists of HNH, RuvC, and PAM interacting domains (Nishimasu et al., 2014). The HNH-like domain of Cas9 cuts the target strands of double strand DNA with the help of PAM interacting domains that confer specificity to PAM recognition sites next to the target site on the DNA sequence (Nishimasu et al., 2014; Mei et al., 2016; Jiang and Doudna, 2017). In contrast, the RuvC-like domain of Cas9 that is structurally similar to proteins of retroviral integrase superfamily defined by an RNase H fold cleaves the non-targeted strand (Ariyoshi et al., 1994). Mutagenesis studies have shown the importance of these two domains in Cas9 DNA endonuclease function and Cas9’s ability to function as an endonuclease is lost when both domains are altered (Nishimasu et al., 2014). Numerous studies on Cas9 orthologs and various PAM variants have greatly benefited our knowledge of CRISPR-Cas9 mechanisms (Fonfara et al., 2014; Nishimasu et al., 2014; Mei et al., 2016; Miller et al., 2020).

## CRISPR/Cas9 based high throughput screens for cancer biomarker discovery

One of the challenges of cancer treatment is its highly heterogeneous nature, which causes drug resistance and relapse cancer cells (Ge et al., 2022). Much progress has been made in identifying new therapeutic targets, biomarkers, and genes responsible for drug resistance using CRISPR/Cas9 genetic screens (Zhou and Yao, 2023). Behan et al. has prioritized the therapeutic target for cancer in 324 human cancer cell lines from 30 cancer types (Behan et al., 2019). Results showed that Werner Syndrome ATP-dependent helicase (WRN) was a top hit and a promising target for microsatellite instability tumors (Behan et al., 2019). Similarly, Tzelepis et al. utilized a custom-design library to detect genetic vulnerabilities of human AML cells (Tzelepis et al., 2016). They identified numerous targets, including Bromodomain-containing Protein 4 (BRD4), Histone Methyltransferase DOT1L (DOT1L), and Multiple endocrine neoplasia type 1 (MEN1) for Acute Myeloid Leukemia (AML) (Tzelepis et al., 2016). The CRISPR loss of function screens help identify several essential genes and biomarkers (Tzelepis et al., 2016). McCleland et al. showed the critical role of the bromodomain and extraterminal (BET) BRD4 in colon cancer proliferation using loss of function CRISPR screen (McCleland et al., 2016). Through transcriptomic and genome analysis, the study found that the long noncoding RNA CCAT1 acts as a potential biomarker to determine the sensitivity of colon cancer patients to BET inhibitors (McCleland et al., 2016). Another whole genome CRISPR screen also identifies the genes that mediate cisplatin resistance (Goodspeed et al., 2019). It revealed that MSH2 promotes cisplatin resistance, and bladder tumors have less MSH2, which leads to poor survival during cisplatin treatment (Goodspeed et al., 2019). Researchers have shown that normal functioning mitochondria were important for Acute lymphoblastic leukemia cell lines to resist the panobinostat drug using genome wide CRISPR/Cas9 screening. Interestingly, higher SIRT1 expression caused Acute lymphoblastic leukemia cell lines more susceptible to panobinostat by activating mitochondrial activity and a cell death pathway linked to mitochondria (Jiang et al., 2022). Lysine-specific demethylase 1 (LSD1 or KDM1A) has emerged as a promising therapeutic target in acute myeloid leukemia. Deb et al. have identified genes including the amino acid sensing arm of mTORC1 that can be targeted with LSD-1 using combinational therapies (Deb et al., 2020). Another study used a similar approach with CRISPR/Cas9 knock-out screen to identify genes in ovarian cancer cells (SKOV3 cell line). In this study by Zhang et al., identified protein-L-isoaspartate (D-aspartate) O-methyltransferase (PCMT1) as an essential driver for anoikis resistance. Interestingly, studies showed that blocking PCMT1 with an antibody significantly reduced the cancer cells’ ability to cell invasion and adhesion (Zhang et al., 2022). However, several genes are identified that need proper validation in clinical trials (Goodspeed et al., 2019).

Since the design of CRISPR knocked out library that has targeted around 18,000 genes with about 64,000 unique guide sequences, Studies have revealed genes that are resistant to vemurafenib in melanoma cells (Shalem et al., 2014). Since then, many studies have been published using loss-of-function screens for identifying drug resistance or lethality (Shalem et al., 2014; Lau et al., 2020). Similarly, CRISPR gain of function libraries was also utilized to identify genes that induce drug resistance (Gautron et al., 2021). Studies have employed transcriptional-activated CRISPR library to screen genes that mediate resistance to BRAF inhibitors in A375 cells and human patient melanoma cell lines (Konermann et al., 2015). Recently, CRISPR libraries have been used *in vivo* (Bi et al., 2021)*.* Studies have identified immune evasion genes and immune inhibitory checkpoints across various cancer models. Also, it showed robust IFN signature is linked with suboptimal response to ICB among renal cell carcinoma or melanoma patients. The research also indicates that classical and nonclassical MHC-I class inhibitory checkpoints promote immune evasion driven by IFN response (Bi et al., 2021). Similarly, Scheidmann et al. have shown that breast cancer metastasis involves tightly regulated stepwise steps (Scheidmann et al., 2022). Also, Blood-borne breast cancer metastasis consists of a series of tightly controlled sequential steps and inhibitors such as PLK1 prevent intravasation of circulating tumor cells (CTC) (Scheidmann et al., 2022). CRISPR/dCAS9 mediated DNA demethylation screens have also been used to identify epigenetic modulations and how these modulations can impact the activation of tumor-associated genes, which lead to cancer progression (Tejedor et al., 2023). Studies found that epigenetic reactivation of RSPO2 is associated with impaired cell proliferation in p53 lacking cancer cell lines (Tejedor et al., 2023). Moreover, epigenetic silencing of RSPO2 converts adenoma to carcinoma (Tejedor et al., 2023).

## Tools for using CRISPR/CAS9 technologies

Several CRISPR technologies are available to assist in designing, delivering, and analyzing CRISPR experiments (Table 2). The first generation of Cas9 protein-based genome editing involves wild-type Cas9 (Tycko et al., 2016). The most popular used Cas9 includes SpCas9 for *Streptococcus pyogenes*. However, some modifications have been made to the wild type Cas9 protein to make it more specific and efficient than before (Kleinstiver et al., 2016; Tycko et al., 2016; Lee et al., 2018). Research has shown that a single mutation with a change from arginine to alanine in wild-type Cas9 leads to a generation of High Fidelity Cas9 (HiFi Cas9) (Vakulskas et al., 2018). HiFi Cas9 has been shown to have reduced off-target effects and high specificity while performing site-specific mutagenesis (Vakulskas et al., 2018). Other variants of Cas9 include eSpCas9 (1.1), which contains mutations at three amino acid K848A/K1003A/R1060A residues (Slaymaker et al., 2016). These mutations provide more specificity to the wild-type Cas9 and weaken the interaction between Cas9 and non-complimentary DNA strands (Nierzwicki et al., 2021). It is found that eSpCas9 (1.1) has 10-fold more specificity at performing site-directed mutagenesis than wild-type Cas9 and exhibits a reduction in off-targeted mutation genome-wide (Kleinstiver et al., 2016). Similarly, SpCas9-HF1 has a quadrupled mutation at different amino acids (N497A/R661A/Q695A/Q926A) (Kleinstiver et al., 2016). Research has shown that mutating non-specific DNA contacts of SpCas9 reduced the off-target effects in human cells and increased its activity by 70 percent greater than wild-type SpCas9 activities for 32 out of 37 of the sgRNA tested (Kleinstiver et al., 2016). Another important component for targeted site-directed mutagenesis is guide RNA (Xu and Li, 2020). Guide RNA should be designed to target the desired genomic sequence, specifically with minimizing the off-target effects (Schneider, 2020). The generation of guide RNA also depends upon the specific applications (Pelea et al., 2022; Shaw et al., 2022). For instance, guide RNAs target constitutively expressed exons and regions at N-terminus for genetic knockouts, which lowers the likelihood that the targeted region will be excised from the mRNA due to alternative splicing and increase the likelihood of frameshift mutations that produce non-functional protein (Mohr et al., 2016). Also, it is essential to target exons that code for essential domains of the protein, so even the introduction of non-frameshift mutations in the crucial domain for protein functions results in the formation of truncated protein (Mohr et al., 2016). Other applications for which guide RNA is generated are activating or repressing target genes using dCas-9 activators or dCas9-repressors (Gilbert et al., 2013; Shalem et al., 2014). For these applications, it is recommended to introduce DNA breaks and insertion/deletion of DNA sequences in the promoter region that drive gene expression (Gilbert et al., 2014). The target region for this type of approach involves targeting the DNA region within 20–200 bp upstream of the transcription start sites and have no off-targets close to the other genes (Gilbert et al., 2014). So, while designing guide RNA, it is essential to consider the intended applications, namely, knock in, knockout, CRISPR activation, and CRISPR interference, and proceed with gRNA synthesis (Gilbert et al., 2013; Sander and Joung, 2014). Several tools available for gRNA design, which are listed below in Table 2.

**TABLE 2:** The table contains information regarding tools used for designing gRNA.

S. No	Tool for designing gRNA	Description
1	Add Gene (www.addgene.org/crispr/)	It helps the researchers to identify relevant tools and resources for their study. It has CRISPR guides, books, Plasmid kits and pooled libraries, which can be used to plan specific experiments
2	Microsoft Research Crispr (https://crispr.ml/)	It provides on-target and off-target prediction through web services. It provides end to end guide for design for CRISPR/Cas9. It is maintained by Broad Institute of MIT and Harvard (Doench et al., 2016; Listgarten et al., 2018)
3	CRISPick (https://portals.broadinstitute.org/gppx/crispick/public)	It is an updated version of GPP sgRNA Design tool that offers streamline sgRNA selection process. It is maintained by Broad Institute (Marx, 2020)
4	E-CRISP (http://www.e-crisp.org/E-CRISP/)	E-CRISP is an online application to create gRNA. To locate gRNA binding locations, it uses a quick indexing approach. It is maintained by the German Cancer Research Center (Heigwer et al., 2014)
5	CHOPCHOP (https://chopchop.cbu.uib.no/)	CHOPCHOP provides sophisticated target selection options. It employs effective sequence alignment techniques to shorten search times and successfully predicts off-target binding of sgRNA (Labun et al., 2021)
6	CRISPRdirect (http://crispr.dbcls.jp/)	It helps researchers to identify target sequences with few off-targets. It incorporates genome sequences of humans, mice, rats, marmosets, pigs, chickens, frogs, zebrafish, Ciona, fruit flies, silkworms, *Caenorhabditis elegans*, Arabidopsis, Sorghum, and budding yeast (Naito et al., 2015)
7	CRISPR-ERA (http://crispr-era.stanford.edu/index.jsp)	It is a genome-wide sgRNA design web tool. Using a database of CRISPRi, it can generate large-scale sgRNAs for gene activation or repression. *E. coli*, *B. subtilis*, *S. cerevisiae*, *C. elegans*, fruit flies, zebrafish, mice, rats, and human genome sequences can be used for generation of sgRNA (Liu et al., 2015)
8	Benchling (https://www.benchling.com/crispr)	It is a great tool that allows visualization, optimization, and annotation of multiple gRNA sequences simultaneously. It uses powerful algorithms to instantly assess off target and on target efficiency of the guides (Uniyal et al., 2019)
9	Synthego CRISPR Design Tool (https://design.synthego.com/#/)	It enables to generate guide RNA for over 120,000 genomes and 9,000 species. It also reduces the time to design guide RNA from hours to minutes (Enzmann and Wronski, 2018)
10	CCTop (https://cctop.cos.uni-heidelberg.de)	It is a CRISPR/Cas9 target online predictor. The tool identifies target sites for the designed sgRNA and based on off-target effects ranks them. It is maintained by the University of Heidelberg (Stemmer et al., 2015)
11	CRISPOR (http://crispor.tefor.net)	It is a web-based tool for designing, assessing and cloning sgRNA sequences. The user is required to provide DNA sequence and genome as input in FASTA format. The tool uses algorithms to predict off target and on-target scores (Concordet and Haeussler, 2018)

## Applications of CRISPR/Cas9 in cancer preclinical studies

Treatment of cancer through the removal of tissues or complete organs, chemotherapy, and radiotherapy is a widely used method to treat cancer (Wang et al., 2018). However, these invasive methods lead to radiation injury and drug toxicity (Wang et al., 2018). CRISPR technology has held promise in research and treatment in various fields, including cancer research and treatment (Xu and Li, 2020). CRISPR technology can be used to modify immune cells, specifically T cells extracted from patients and transferred back to patients (Stadtmauer et al., 2020). These CRISPR-modified immune cells have the capability to detect and remove any abnormal cells or cancer cells (Stadtmauer et al., 2020). Studies have shown that cancer cells can evade the immune system’s ability to detect and suppress cell growth, which leads to uncontrolled tumor growth (Sica et al., 2008). Using CRISPR technology helps overcome these barriers and boost the immune response against cancer (Zhan et al., 2019). CRISPR/Cas9 is used to delete particular genes or mutate associated with cancer (Zhan et al., 2019). By using CRISPR/Cas9, it has been shown that CD133 has been knocked out, leading to the downregulation of vimentin expression in colon cancer cells (Li et al., 2019). As a result, there has been a significant reduction in cell proliferation and colony formation, along with a notable decrease in cell migration and invasion (Li et al., 2019). miR-3064 is crucial for pancreatic cancer and acts as a tumor suppressor (Yan et al., 2019). It is well established that miR-3064 promotes pancreatic cancer growth, invasion, clone creation, and sphere formation (Yan et al., 2019). However, CRISPR/Cas9-mediated knockout of miR-3064 reverts these malignant processes (Yan et al., 2019). Similarly, Focal Adhesion Kinase (FAK) is a multifunctional protein regulator of DNA damage repair in mutant KRAS NSCLC and dysregulation of FAK has implications for cancer progression (Tang et al., 2016). NSCLC cells with KRAS mutations showed detectable DNA damage and increased sensitivity to radiotherapy upon FAK gene knockdown using CRISPR/Cas9 (Tang et al., 2016).

Ribonucleotide reductase (RRM1) is an important enzyme that catalyzes the rate-limiting step in the formation of deoxyribonucleosides (Cory and Sato, 1983). The studies have shown that conditional deletion of RRM1 in Ewing sarcoma cells causes the increase in expression of genes like c-Jun and c-Fos that hinder tumor growth and progression (Croushore et al., 2023). An increase in nucleotide biosynthesis in cancerous cells is also one of the factors responsible for increased cell proliferation (Villa et al., 2019). The study has shown that CRISPR-mediated depletion of ubiquitin-specific peptidase 29 (USP29) leads to the disruption of intermediates accountable for involvement in glycolysis and nucleotide biosynthesis in neuroblastoma cell lines (Chandrasekaran et al., 2021). USP29 has also been shown to promote neuroblastoma progression by upregulating glycolysis and glutamine catabolism (Kang et al., 2023). The study has also been conducted to target ferroptosis as an attractive strategy in cancer therapy (Alborzinia et al., 2023). LRP8 is a selenoprotein P receptor that is important for protecting MYCN-amplified neuroblastoma (Alborzinia et al., 2023). Using CRISPR/Cas9, LRP8 has been genetically deleted, resulting in the depletion of selenocysteine required for translation of anti-ferroptosis GPX4 and making MYCN-amplified neuroblastoma vulnerable to cell death (Alborzinia et al., 2023). PUM1 protein can regulate dead-box helicase 5 (DDX5), which increases cell viability and proliferation (Liu Q. et al., 2021). Liu et al. have shown that CRISPR-mediated knockdown of PUM1 and DDX5 will lead to decreased tumor cell viability (Liu Q. et al., 2021). Similarly, the HMGA2-WHSC1 axis regulates cancer cell growth, proliferation, and metastasis, where WHSC1 acts as a transcription factor for oncogene HMGA2 (Liu H. H. et al., 2021). Liu et al. have demonstrated using CRISPR/Cas9 that WHSC1 inhibits colon cancer cell proliferation, boosts drug sensitivity, and reduces metastatic ability in colon cancer cells (Liu H. H. et al., 2021). Cycle-dependent kinase p38γ (p38γ) is highly expressed in colon cancer and is involved in tumor growth and migration (Su et al., 2019). Su et al. showed that p38γ deletion results in cell proliferation and growth (Su et al., 2019). In case of colorectal adenocarcinoma, increased expression of Aldoase B is linked to a poor prognosis and accelerates tumor growth (Li et al., 2017). In cancer cells (LoVo and SW480), knockout of Aldolase B using CRISPR demonstrated that Aldolase B inhibits proliferation, migration, and invasion in these cancer cells (Li et al., 2017). RhoV is a key driver gene associated with and is upregulated in triple-negative breast cancer (Jin et al., 2023). Studies have shown *in vivo* functional screens identified RhoV as a regulator of tumor metastasis (Jin et al., 2023). Jin et al. have demonstrated that the knockout of RhoV suppressed cell invasion, migration, and metastasis (Jin et al., 2023). Additionally, it provides evidence that RhoV interacts with p-EGFR to activate the downstream signaling (Jin et al., 2023). Cell division cycle 7 (CDC7) was identified by Deng. et al. using CRISPR/Cas9 screening (Deng et al., 2023). In chemo-resistant small cell lung cancer, CDC7 possibly acts as synergistic target (Deng et al., 2023). Studies revealed that suppressing CDC7 lowered the IC50 and increased chemotherapy effectiveness in chemo-resistant SCLC cells (Deng et al., 2023). Another CRISPR/Cas9 screen has identified a Zinc transporter (ZIP9) and using CRISPR-mediated ZIP9 deletion showed that knockout of ZIP9 causes dysregulation of Zinc homeostasis, which is associated with N-terminal linked glycosylation resulting in cancer-like glycosylation on the surface of the cell surface (Wang et al., 2020). Tissue inhibitor of metalloproteinase-2 (TIMP-2) has a role in remodeling the extracellular matrix to promote cancer progression (Escalona et al., 2022). Escalona et al. have shown CRISPR/Cas9-mediated depletion of TIMP-2 in ovarian cell cancer leads to inhibition of cell growth, migration, invasion, and proliferation (Escalona et al., 2022). There are many studies available that show importance of CRISPR in treatment of cancer. However, due to limited space, we have discussed recent studies only.

## CRISPR usage in clinical cancer studies

In CAR-T immunotherapy, CRISPR-mediated genetically altered T cells are used to detect cancer cells (Jogalekar et al., 2022). These T cells strategically target cancer cells and eliminate them from the body (Jogalekar et al., 2022). The FDA approved CAR-T for treating patients with traditional gene therapy in 2017 (Chen et al., 2023). Moreover, researchers have been working on making more powerful CAR-T therapies using T cells from a healthy donor (Chen et al., 2023). These T cells are allogeneic in nature and edited to attack cancer cells (Chen et al., 2023). Further, these T cells avoid the recipient’s immune system or do not trigger graft vs. host response (Sanber et al., 2021).

CD70 is a CD27 receptor ligand protein, a cell surface protein with transitory expression on activated lymphocytes (Hintzen et al., 1995) and has increased expression in clear cell RCC (Jilaveanu et al., 2012). CTX130 is the first CAR T-cell therapy used in patients targeting CD70 (Pal et al., 2022). It has been demonstrated that the gene-edited allogeneic CAR-T used in CTX130 treatment is directed against the novel target CD70, which can cause relapsed or refractory T-cell lymphomas in patients (Pal et al., 2022). The phase 1 COBALT-RCC trial (NCT04438083) data from 13 patients showed that the objective response rate was 8%, rate of stable disease was 69%, and rate of disease control was 77% (Pal et al., 2022). It is also observed that therapy has an acceptable safety profile except three patients have severe adverse events and one death unrelated to CTX130 therapy (Pal et al., 2022). In another first phase studies, Stadtmauer et al. assess the viability and security of employing CRISPR/Cas9 to modify T cells (Stadtmauer et al., 2020). In the study, three cancer patients with refractory cancer participated in the trial (Stadtmauer et al., 2020). It is observed that there is a reduction in TCR mispairing and increased production of a cancer specific TCR transgenic with CRISPR/Cas9 mediated deletion of genes in charge of indigenous T cell receptor (TCR) genes (Stadtmauer et al., 2020). They also remove a gene that encoding programmed cell death protein 1 (PD-1) to enhance antitumor immunity (Stadtmauer et al., 2020). The CRISPR/Cas9 system’s holds promise for gene-edited immunotherapies, as evidenced by the fact that all three T cell transfers were effective and persisted for up to 9 months (Stadtmauer et al., 2020). Wang and others identified 15 patients with solid mesothelin-positive tumors (Wang et al., 2021). Mesothelin-specific CAR-T cells lacking PD-1 and TCRs are generated using CRISPR/Cas9 and then evaluated with increased dosage of the drug (Wang et al., 2021). The findings demonstrated that two patients had stable illnesses, and the circulation of altered T cells peaked between days 7 and 14 (Wang et al., 2021). After a month, the edited T cells were undetectable and had no toxicities or severe side effects (Wang et al., 2021). This study provides more evidence of the viability and safety of T cells altered by CRISPR/Cas9 (Wang et al., 2021). In a recent study, Liao and others have shown that CRISPR/Cas9 can be used to knockout PD-L1, a potential target, in patients suffering from osteosarcoma (Liao et al., 2017). This breakthrough discovery marks the initial stages of establishing the safety and effectiveness of CRISPR/Cas9 for treating other malignancies, including NSCLC and sarcoma (Liao et al., 2017). It is particularly significant due to the crucial role of the PD-1/PD-L1 axis in cancer immune escape and therapeutics. In other phase I trial, 16 patients with varied different resistant solid tumors were treated by Foy et al. using CRISPR/Cas9 technology by knocking out two T cell receptor genes (Foy et al., 2023). Each participating patient in a clinical trial received up to three edited modified TCR products in dose escalation (Foy et al., 2023). Neurotoxicity or the cytokine release syndrome only occurred in only two cases. The best therapeutic response in five patients was stable illness, demonstrating the viability of isolating endogenous T cell receptors and using CRISPR/Cas9’s simultaneous knockout and knock-in technology (Foy et al., 2023). In another phase I trial, XFF19 CAR-T cells are autologous T cells designed to target CD19 and CRISPR gene altered to remove endogenous HPK1 in CD19^+^ leukemia or lymphoma (NCT04037566). Also, CTX110 and CTX112 are also used against relapsed or refractory B-cell malignancies and target CD19 (NCT04035434, NCT05643742). Similarly, CTX120 therapy is used in patients with relapsed or resistant multiple myeloma and target B-cell maturation antigen (BCMA)(NCT042446560). Phase I trials for PD-1 targets in EBV-associated malignancies, phase II trials for CD19^+^ leukemia and lymphoma, relapsed or refractory leukemia and lymphoma, and advanced esophageal cancer are just a few of the numerous clinical trials that are now being conducted (Table 3).

**TABLE 3:** Recent clinical trials conducted to combat cancer using CRISPR technology.

S. No	ClinicalTrails.gov ID	Cancer associated	Therapy	Reference
1	NCT03970382	Solid Tumors	Gene edited autologous autologous NeoTCR-T cells administrated with or without Anti-PD1	Foy et al. (2023)
2	NCT04438083	Refractory Renal Cell Carcinoma	CTX130 (CD70-directed T-cell immunotherapy comprised of allogeneic T cells genetically modified *ex vivo*)	Pal et al. (2022)
3	NCT04426669	Metastatic gastrointestinal cancers	Knockout of intracellular immune checkpoint in Tumor infiltrating lymphocytes	Palmer et al. (2020)
4	NCT04637763	Refractory B cell Non-Hodgkin lymphoma	Allogenic anti CD-19 CAR-T cell	O'Brien et al. (2022)
5	NCT05722418	Multiple myeloma	Anti-BCMA CAR-T cell therapy	Berdeja et al. (2023)
6	NCT04037566	All Leukemia and Lymphoma	HPK-1 knockout CD19-specific CAR-T cells	Si et al. (2020)
7	NCT04035434	Refractory B-Cell Malignancies	CTX110 (CD70-directed T-cell immunotherapy comprised of allogeneic T cells genetically modified *ex vivo*)	McGuirk et al. (2021)
8	NCT05037669	All leukemia and lymphoma	Targeted gene: TCR, HLA-class I and HLA-class II	Lan et al. (2022)
9	NCT03545815	Mesothelin positive multiple solid tumors	Knock out PD-1 and TCR gene in mesothelin directed CAR-T cells	Wang et al. (2021)
10	NCT02793856	Metastatic non-small cell lung cancer	Knock out PD-1 T cells	Lu et al. (2020)

## CRISPR/Cas9 in cancer drug screening

**FIGURE 2:**
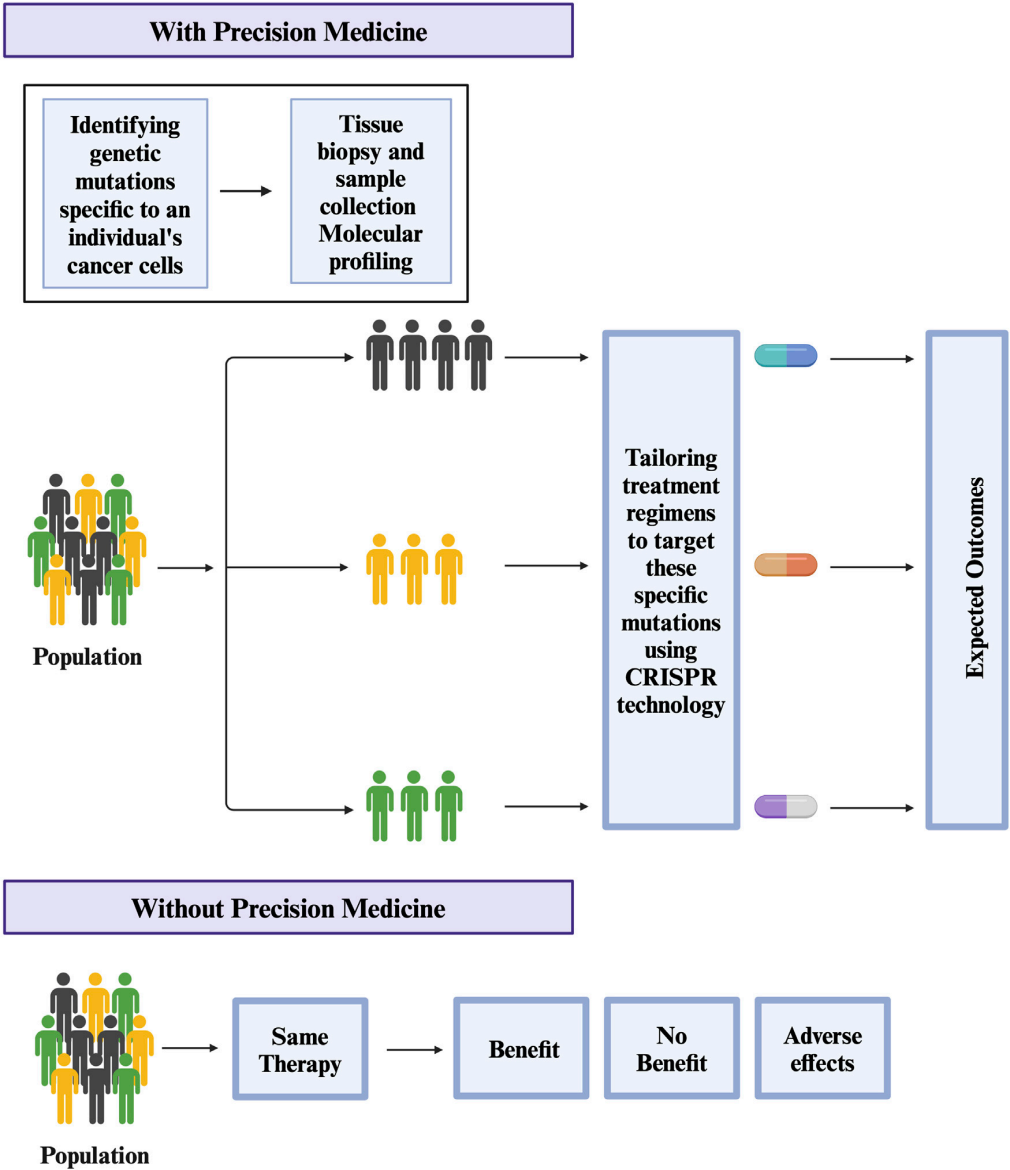
Potential use of CRISPR technology in Precision medicine for treating Cancer.

Cancer tumors are heterogeneous and regulated by numerous genes (Sun and Yu, 2015). To understand the role of multiple genes in cancer, researchers are using a combinatorial CRISPR/Cas9 approach in which multiple guide RNAs are used to knock out multiple regulatory genes (Han et al., 2017; Giri et al., 2019; Ianevski et al., 2019; Ianevski et al., 2021). One example of this approach is a CRISPR-based double knockout (CDKO) system that was used to generate a large-scale human gene interaction (GI) map (Han et al., 2017). This system used 490,000 double-sgRNAs directed against 21,321 pairs of drug targets in K562 leukemia cells (Han et al., 2017). This large-scale map can be used to develop more personalized targeted therapies in the future (Figure 2). Another example is a study by Najm et al. who used a combinatorial screening approach to explore complex gene networks. The study used machine learning to pair *S. aureus* Cas9 with sgRNA to identify synthetic lethality and gene pairs across multiple cell types (Najm et al., 2018). These combinatorics CRISPR libraries are commonly used to study genes responsible for drug resistance (Najm et al., 2018). Recently, CRISPR library screens can also be combined with other techniques to understand different pathways in cancer (Romero et al., 2017). For instance, Replogle et al. combined CRISPR/Cas9-based genetic screening and metabolomic analyses to show that Keap1/Nrf2-mutant cancers are dependent on increased glutaminolysis, and this key insight can be therapeutically exploited for cancer treatment (Romero et al., 2017). Similarly, brain tumors are used to identify signaling networks downstream of cancer driver genes. By combining whole proteome, phosphoproteome, transcriptome, and systems biology approaches, researchers have identified numerous master regulators, including 41 kinases and 23 transcription factors (Wang et al., 2019). In the same study, validation studies have shown that more than 50 percent of master regulators are important for cancer growth and novel tumor vulnerabilities (Wang et al., 2019). GWAS studies have also led to the development of new treatments for cancer and other diseases (Giri et al., 2014; Prasad et al., 2019a; Prasad et al., 2019b). For example, GWAS studies have identified genetic variants that are associated with resistance to certain chemotherapy drugs in cancer (Innocenti et al., 2011; Pützer et al., 2020). Overall, these development helps investigators to identify new drug targets, validate drug targets, understand mechanisms of drug resistance, and develop more personalized targeted therapies.

## Limitations of CRISPR/Cas9 technology and future promise

CRISPR/Cas9 is a powerful tool to edit the DNA genome. However, it has limitations, namely off-target effects, elicits of the host immune system against endogenous Cas9 proteins and moral concerns of gene editing (Rasul et al., 2022). The off-targeting effects of CRISPR/Cas9 involve binding of gRNA at undesired locations in the genome and causing cleavage using Cas9 protein to generate harmful mutations (Rasul et al., 2022). According to studies, the number of off-target sites varied from 10 to >1,000, depending on the gRNA (Tycko et al., 2016). Studies have shown the importance of the PAM binding site of sgRNA and cas9 binding site (Tycko et al., 2016). However, strategies have been developed that showed better bioinformatics tools, modified Cas9 nickases, and anti-CRISPR proteins cause less off-target effect using CRISPR/Cas9 protein (Guo et al., 2023). Another problem with the CRISPR/Cas9 system is that Cas9 is a bacterial protein that can elicit a host immune response (Crudele and Chamberlain, 2018). The host immune system recognizes Cas9 as the foreign protein that leads to the degradation of these proteins, which prevents it from performing its function (Crudele and Chamberlain, 2018). Next, there are ethical concerns regarding the use of CRISPR/Cas9, as CRISPR can be used for human eugenics (Ayanoglu et al., 2020). Editing the genes of embryos or germline cells can lead to permanent genetic changes that are passed down to future generations (Ayanoglu et al., 2020). Manipulating the genes can have unforeseen results, leading to potential increased susceptibility to other diseases (Ayanoglu et al., 2020). While CRISPR has immense promise for cancer precision, it faces hurdles beyond technical limitations and immune toxicity concerns. Cancer’s heterogeneity is a major challenge. Different tumors within the same cancer type in different individual patients can have unique genetic and epigenetic changes. Targeting specific genetic drivers of each patient’s cancer using CRISPR technology will be difficult because of the intricate interplay between various cellular processes and signaling pathways. Additionally, CRISPR editing can sometimes lead to mosaic mutations, where only a portion of tumor cells receive the desired change (Mehravar et al., 2019). This heterogeneity within the tumor can hinder the effectiveness of CRISPR therapies and contribute to resistance.

In conclusion, CRISPR/Cas9 has emerged as a promising tool in the battle against cancer. CRISPR is a revolutionary gene-editing technology that offers unparalleled precision in targeting and modifying cancer-related genes. The CRISPR’s ability to edit the genes and disrupt genes responsible for tumorigenesis and metastasis will open new avenues for developing personalized, effective, and less toxic patient treatments. While the field of CRISPR/Cas9 therapeutics for cancer treatment is still nascent, the results from preclinical studies and clinical trials are promising. These developments bring hope to develop more targeted and less invasive interventions for cancer treatment.
